# UV-B Radiation Affects Photosynthesis-Related Processes of Two Italian *Olea europaea* (L.) Varieties Differently

**DOI:** 10.3390/plants9121712

**Published:** 2020-12-04

**Authors:** Chiara Piccini, Giampiero Cai, Maria Celeste Dias, Marco Romi, Roberta Longo, Claudio Cantini

**Affiliations:** 1 Department of Life Sciences, University of Siena, via Mattioli 4, 53100 Siena, Italy; cai@unisi.it (G.C.); marco.romi@unisi.it (M.R.); roberta.longo@student.unisi.it (R.L.); 2 Institute for BioEconomy, National Research Council of Italy, 58022 Follonica, Italy; claudio.cantini@ibe.cnr.it; 3 Department of Life Sciences, Centre for Functional Ecology, University of Coimbra, Calçada Martim de Freitas, 3000-456 Coimbra, Portugal; celeste.dias@uc.pt

**Keywords:** UV-B radiation, *Olea europae*, photosynthesis, pigments, sugars, antioxidant capacity

## Abstract

Given the economical importance of the olive tree it is essential to study its responses to stress agents such as excessive UV-B radiation, to understand the defense mechanisms and to identify the varieties that are able to cope with it. In the light of the analysis carried out in this study, we argue that UV-B radiation represents a dangerous source of stress for the olive tree, especially in the current increasingly changing environmental conditions. Both the varieties considered (Giarraffa and Olivastra Seggianese), although resistant to the strong treatment to which they were exposed, showed, albeit in different ways and at different times, evident effects. The two varieties have different response times and the Giarraffa variety seems better suited to prolonged UV-B stress, possible due to a more efficient and quick activation of the antioxidant response (e.g., flavonoids use to counteract reactive oxygen species) and because of its capacity to maintain the photosynthetic efficiency as well as a relatively higher content of mannitol. Moreover, pigments reduction after a long period of UV-B exposure can also be an adaptation mechanism triggered by Giarraffa to reduce energy absorption under UV-B stress. Olivastra Seggianese seems less suited to overcome UV-B stress for a long period (e.g., higher reduction of *F_v_*/*F_m_*) and has a higher requirement for sugars (e.g., glucose) possible to counteract stress and to restore energy.

## 1. Introduction

One of the issues intricately linked to climate change is the reduction of the ozone layer, the latter being significantly increased by air pollution [1]. In fact, the stratospheric ozone shields 90% of UV-B radiation [2], therefore its degradation causes a higher exposure of organisms to this radiation and increases the risks connected to UV-B exposure [3]. Excessive exposure to UV-B radiation exerts adverse impacts of different types, which include a wide range of morphological, physiological and reproductive aspects on plants, animals, and humans [4]. The awareness of the dangerous effects of ozone layer reduction gave rise to several changes at the global level (e.g., implementation of the Montreal Protocol) resulting in the slowdown of the ozone layer depletion, but ozone layer is still lower than in the pre-1980 era. Currently, the levels of UV-B reaching the earth surface vary considerably, reaching values around 15 KJ m^−2^ d^−1^ in the Mediterranean basin or even extremely higher values, around 65 KJ m^−2^ d^−1^, in Lhasa (Tibet) during the summer [5]. Nevertheless, elevated UV-B levels are expected to continue over the 21st century, particularly in regions were clear sky is a typical condition [6].

UV-B radiation (280–315 nm) play an important role in terrestrial ecosystems but, when in excess, it can represent a risk for plants, inducing numerous negative effects, both direct and indirect, to which plants can respond with defense and adaptation mechanisms, depending on the species and the environmental conditions in which they live [7]. DNA is one of the macromolecules most at risk of UV radiation; specifically, UV-B radiation can cause gene mutations triggering the production of cyclobutane-pyrimidine dimers and, to a lesser extent, pyrimidine (6-4) -pyrimidinone (6-4 PP) dimers [8]. In addition to being mutagenic, both RNA and DNA polymerase are unable to read unrepaired dimers, leading to a blockage in gene transcription and DNA replication [8]. Furthermore, studies have shown the presence of oxidative DNA lesions induced by UV rays in plants [9]. In fact, exposure of plant tissues to UV-B rays increases the production of reactive oxygen species (ROS) causing damage to nucleic acids as well as to proteins and lipids [10].

Another main target of UV-B radiation is the photosynthetic apparatus, which is particularly sensitive to UV-B exposure [9]. High UV-B radiation results in a decrease in photosynthetic efficiency, reduction of the growth rate, and alterations in the metabolism of carbon and nitrogen [9,10]. UV-B radiation can also affect stomatal conductance, altering the rate of water loss through transpiration and net CO_2_ assimilation rate [9,11]. Studies on direct injuries of the photosynthetic apparatus by UV-B radiation showed inactivation of photosystem II (PSII) [9,12], decrease in photosynthetic pigment levels [13], alteration of the integrity of thylakoids and chloroplast ultrastructure [9], reduction in the activity of Rubisco [14,15] and down-regulation of transcription of photosynthetic genes [16]. UV-B rays can also cause photomorphogenic modifications and damages, especially in leaves. Curling of leaves, for example, aims at reducing the surface exposed to radiation [17]. It has also been observed that the increase in UV-B radiation in several species reduces the height of plants, the leaf fresh mass and area, the production of total biomass, and changes the morphology of leaves [18,19]. Negative effects of UV-B radiation can also be extended to other plant processes, including the reproductive ones. In some cases, alterations of the reproductive system have been observed as a result of decrease in pollen germination [20]. For instance, in the olive tree, both pollen germination and pollen tube length were strongly reduced by high levels of UV-B [11].

Although plants have developed numerous repair and protection mechanisms over time, the damage caused by UV-B radiations is still significant [7]. Among the defense mechanisms that plants activate in response to UV-B stress, the enzymatic and non-enzymatic mechanisms that counteract the generation of ROS and their subsequent reactions are of extreme importance. Antioxidants include enzymes like superoxide dismutase, catalase and the Halliwell/Asada pathway enzymes, as well as non-enzymatic substances like glutathione, ascorbate, tocopherols, carotenoids, albumin, bilirubin, chelating agents and phenolics [7,12,21]. With special reference to phenolic compounds, flavonoids are reported to effectively absorb UV-B radiation and to neutralize reactive oxygen species (ROS) [22]. Furthermore, exposure to UV-B radiation can increase the concentration of other phenolic compounds such as the hydrocinnamic acids and secoiridoids that can also efficiently protect plants against the deleterious effects of UV-B stress [23].

The Mediterranean region is one of the most vulnerable to climate change and the negative effects of the high levels of UV-B radiation on several typical species of this region has already been highlighted (e.g., [15,24,25]). Olive trees (*Olea europaea* L.) are one of the most important and oldest crops in the Mediterranean basin. Despite the high adaption of this species to the environmental condition of Mediterranean region, the continuous elevated levels of UV-B radiation predicted for the near future (e.g., [26,27]) together with other multiple environmental factors characteristics of these region (such as sky cloudiness and high air pollutants) represent a risk to olive culture and productivity, as already highlighted in other reports [15,23,28]. It is therefore urgent understand how elevated UV-B radiation affects olive plants physiology and identify varieties better adapt to these conditions, allowing farmers to grow selected varieties that are suitable for the current and future environmental situation. In this study we aimed at integrating the contribution of previous surveys [15,23,29,30], studying the physiological response, particularly photosynthesis, pigments, carbohydrates and antioxidant compounds, of two Italian Olea europaea varieties (Olivastra Seggianese typical from the Toscana region and Giarraffa from Sicilia region) to high levels of UV-B radiation. The Olivastra Seggianese is a variety widespread only in its area of origin: on the slopes of Monte Amiata in Tuscany. The plants of this cultivar reach considerable size. The fruits are small in size with a spherical shape and ripen early and simultaneously. The quantity of oil in the olives is high and of good quality. It is a hardy plant that resists low temperatures. Giarraffa, on the other hand, is cultivated in many areas of Sicily but is also found in some areas of Calabria and Puglia. The trees are of medium height. The fruits are quite large, ovoid, ripen early and can also be used for oil extraction. This cultivar shows a low rusticity and a greater susceptibility to attacks by common animal parasites. It has a medium tolerance to low temperatures. In the present study we have investigated these two olive varieties, which are exposed to a different solar radiation because of geographical reasons, in order to evaluate the effects of chronic UV-B stress (14 h per day for 8 weeks) by comparing the sensitivity/tolerance of the two varieties and identifying the most critical time points and the olive plants response adaptations.

## 2. Results

The UV-B treatment was carried out for a period of 8 weeks for 14 h a day. The treatment scheme was performed according to Nogués and Baker [24]. During the treatment eight time points were established: the first one before the onset of UV-B treatment (T0), after 1, 2, 3, 4, 5, 6, 7 and 8 weeks of UV-B treatment (indicated respectively as T1, T2, T3, T4, T5, T6, T7 and T8). Photosynthetic efficiency was measure in fresh material in all time points (in control and UV-B treated plants), since it is an indicator of the plant photosynthetic performance and it is a non-destructive parameter. Additionally, leaf samples were collected in five representative sampling times (T0, T2, T4, T6 and T8), immediately frozen in liquid nitrogen and stored at −80 °C.

### 2.1. Photosynthetic Efficiency

**Figure 1:**
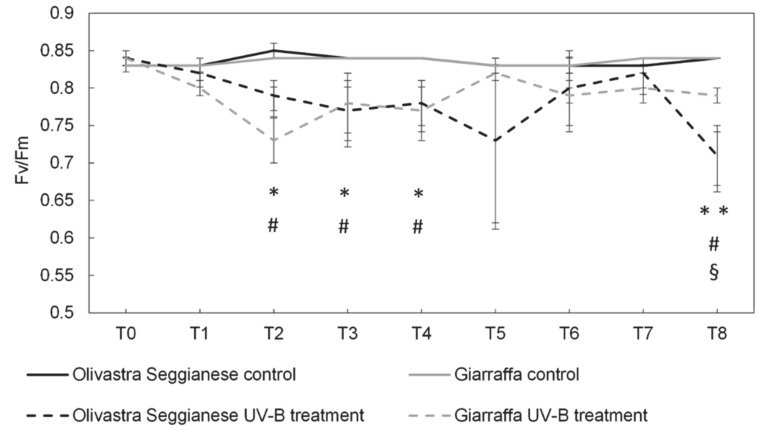
On the x-axes the time points, on the y-axes the *F_v_*/*F_m_* value. Maximum photochemical efficiency (*F_v_*/*F_m_*) in the two olive varieties, Olivastra Seggianese and Giarraffa, under control and UV-B treatment in the different sampling times. In each line, values are given as mean ± standard deviation. Asterisk (*) represent significant differences between control and treated plants of Olivastra Seggianese (* *p* ≤ 0.05; ** *p* ≤ 0.01). Hashtag (#) represent significant differences between control and treated plants of Giarraffa (*p* ≤ 0.05). The § symbol represent significant differences between Olivastra Seggianese treated plants and Giarraffa treated plants (*p* ≤ 0.05).

The *F_v_*/*F_m_* in the control plants of both varieties did not differ significantly (confirmed by ANOVA test) as the response trend of both varieties overlap over time (Figure 1).

The *F_v_*/*F_m_* values in control plants ranged from 0.83 to 0.84 all over the experiment. However, more discrepancies were evident when comparing the trend of plants subjected to UV-B with those of control.

As expected, before the onset of stress (T0), all plants (control and those undergoing UV-B stress) have similar *F_v_*/*F_m_* values. After the first week of stress (T1), control plants and UV-B plants from the variety Olivastra Seggianese showed similar (*p* > 0.05) *F_v_*/*F_m_* averages. However, comparing both varieties under UV-B conditions, the *F_v_*/*F_m_* in Olivastra Seggianese was significantly higher than the one of Giarrafa (0.82 ± 0.01 and 0.80 ± 0.01, respectively). At T2, it is possible to observe a remarkable decrease (*p* ≤ 0.05) in the *F_v_*/*F_m_* ratio in UV-B treated plants when compared with the controls, for both varieties. At this point the *F_v_*/*F_m_* was around 0.79 in UV-B Olivastra Seggianese plants and 0.73 in UV-B Giarraffa plants. From T3 to T4, UV-B treated plants of both varieties showed a lower value of *F_v_*/*F_m_* (*p* ≤ 0.05) than control, but no significant differences were found in both varieties treated with UV-B. At T5, the UV-B Olivastra Seggianese reported slightly higher fluctuations with a sharp decrease, but this was not statistically significant (compared to UV-B Giarraffa and control plants). In the next sampling points (T6 and T7), control and UV-B plants showed similar (*p* > 0.05) *F_v_*/*F_m_* values. At T8, UV-B-stressed Olivastra Seggianese showed a decrease (*p* ≤ 0.01) in the *F_v_*/*F_m_* to values around 0.71, while controls remain stable. UV-B Giarraffa plants were still in a plateau phase, but with an *F_v_*/*F_m_* significantly lower (*p* ≤ 0.05) than the control. At this time UV-B plants of the two varieties showed a statistically significant difference (*p* ≤ 0.05).

**Figure 2:**
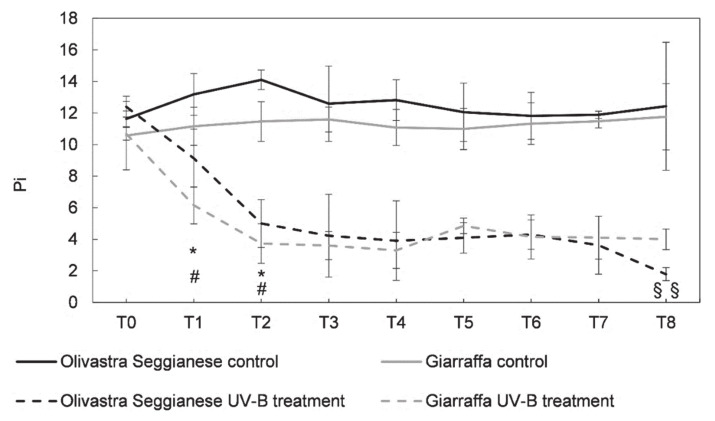
On the x-axes the time points, on the y-axes the Pi value. Performance index (Pi) in the two olive varieties, Olivastra Seggianese and Giarraffa, under control and UV-B treatment in the different sampling times. In each line, values are given as mean ± standard deviation. Asterisk (*) represent significant differences between control and treated plants of Olivastra Seggianese (*p* ≤ 0.05). Hashtag (#) represent significant differences between control and treated plants of Giarraffa (*p* ≤ 0.05). Double §§ represent significant differences between Olivastra Seggianese treated plants and Giarraffa treated plants (*p* ≤ 0.01).

As specified in the method section, the performance Index (Pi) is a more sensitive parameter indicating the possible variations of the entire photosynthetic apparatus, including photosystems I (PSI) and II (PSII). Concerning the performance index (Pi), the controls of both varieties have a similar trend of response (Figure 2), and do not differ significantly as confirmed by the ANOVA test. Contrarily, the Pi of UV-B plants, which are remarkably similar to those of controls at time point 0, are clearly affected by a decrease (*p* ≤ 0.05) just after one week of stress (T1). At time point 1, controls of Olivastra Seggianese had a Pi around 13.18, while UV-B plants had 9.14. For the control plant of Giarraffa the Pi was around 11.16, while in UV-B plants was 6.15.

After the T2, the Pi decreased significantly (*p* ≤ 0.05) in UV-B plants. While the average Pi in control plants of Olivastra Seggianese is 14.1, in UV-B plants was approximately 5 and in UV-B Giarraffa was approximately 3.73 at T2 (against a value of 11.46 in control plants). Subsequently, from T3 onward, UV-B plants of both varieties enter a plateau phase which persisted up to T7. After a further week of stress (T8), the Pi of the UV-B Olivastra Seggianese plants were affected by a significant drop (1.79 against 12.43 of control plants). The Pi in UV-B Giarraffa plants persist in the plateau phase, with an average Pi value lower than that of controls. At this time point, differences between the mean value of stressed plants of both varieties were statistically significant (*p* ≤ 0.01).

### 2.2. Photosynthetic Pigments

**Figure 3:**
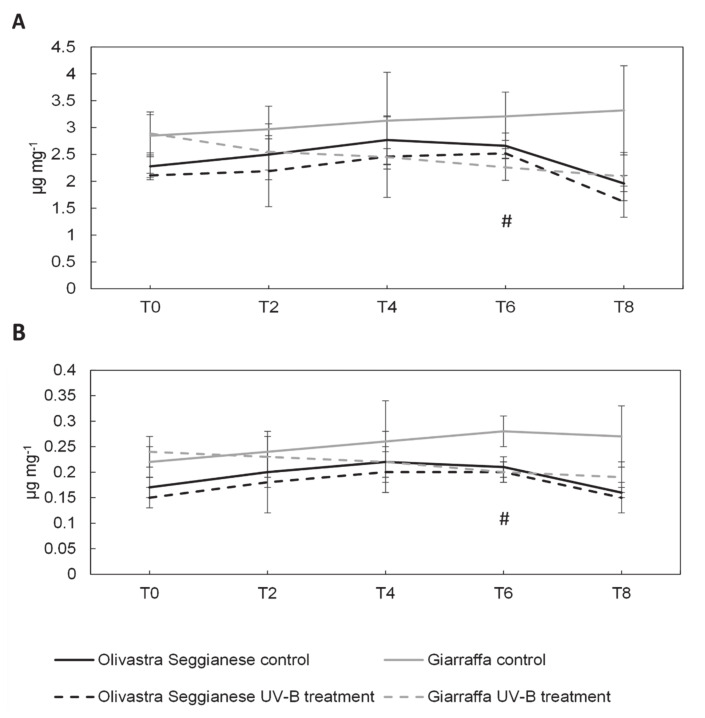
On the x-axes the time points, on the y-axes the concentration of pigments expressed in µg mg^−1^. (**A**) Chlorophyll a content in the two olive varieties, Olivastra Seggianese and Giarraffa, under control and UV-B treatment in the different sampling times. For each column, values are given as mean ± standard deviation. Hashtag (#) represent significant differences between control and treated plants of Giarraffa (*p* ≤ 0.05). (**B**) Chlorophyll b content in the two olive varieties, Olivastra Seggianese and Giarraffa, under control and UV-B treatment in the different sampling times. For each column, values are given as mean ± standard deviation. Hashtag (#) represent significant differences between control and treated plants of Giarraffa (*p* ≤ 0.05).

**Figure 4:**
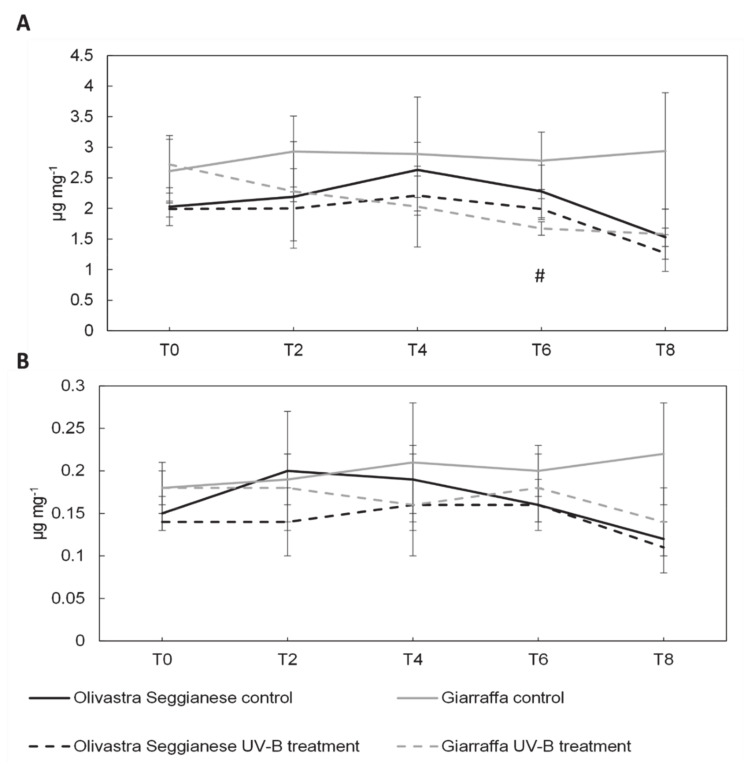
On the x-axes the time points, on the y-axes the concentration of pigments expressed in µg mg^−1^. (**A**) β-carotene content in the two olive varieties, Olivastra Seggianese and Giarraffa, under control and UV-B treatment in the different sampling times. For each column, values are given as mean ± standard deviation. Hashtag (#) represent significant differences between control and treated plants of Giarraffa (*p* ≤ 0.05). (**B**) Lutein content in the two olive varieties, Olivastra Seggianese and Giarraffa, under control and UV-B treatment in the different sampling times. For each column, values are given as mean ± standard deviation.

Figure 3 and Figure 4 show the content of pigments in olive leaves of both varieties (control and UV-B treated plants). The content of chlorophyll a, chlorophyll b, β-carotene and lutein during the sampling times was similar (*p* > 0.05) in control and UV-B Olivastra Seggianese plants. Although the absolute quantity is slightly lower in treated plants than in control ones, the observed difference is inherent in the experimental variation. Therefore, we assumed no statistically significant differences in Olivastra Seggianese between controls and treated plants (also confirmed by ANOVA test). 

Also, the content of these pigments in control and UV-B Giarraffa plants was similar (*p* > 0.05), except at time point 6 where the levels of chlorophyll a, chlorophyll b and β-carotene were higher (*p* ≤ 0.05) in control plants. Even in this case, the ANOVA test did not show significant differences between control and treated plants.

### 2.3. Sugars

**Figure 5:**
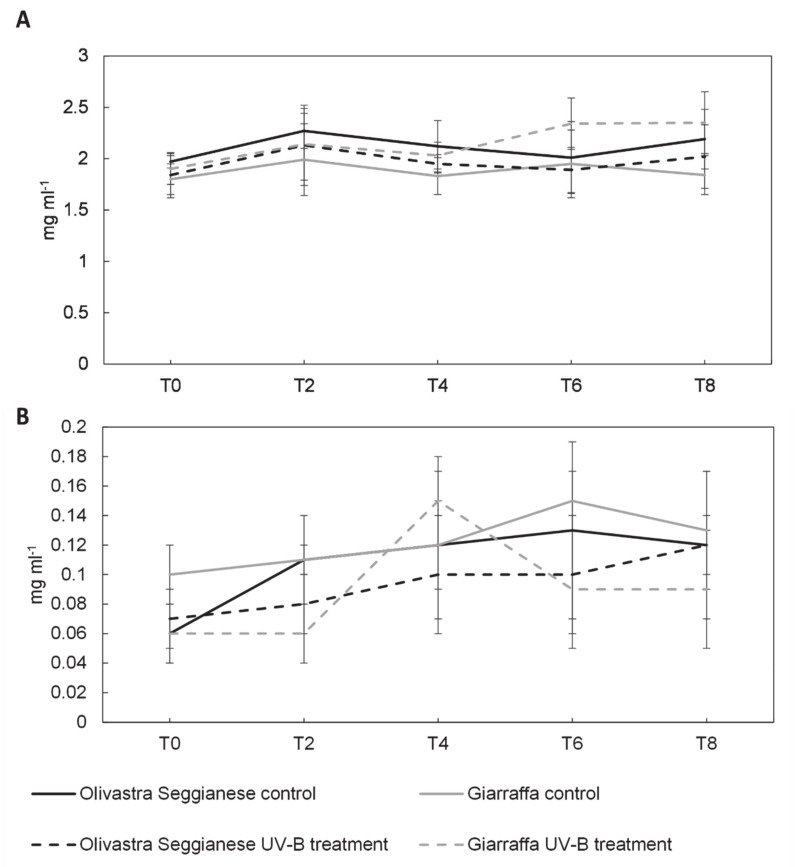
On the x-axes the time points, on the y-axes the concentration of sugars expressed in mg mL^−1^. (**A**) 6-P glucose content in the two olive varieties, Olivastra Seggianese and Giarraffa, under control and UV-B treatment in the different sampling times. For each column, values are given as mean ± standard deviation. (**B**) Sucrose content in the two olive varieties, Olivastra Seggianese and Giarraffa, under control and UV-B treatment in the different sampling times. For each column, values are given as mean ± standard deviation.

At T0 the content of sucrose, glucose 6-P, glucose, fructose and mannitol was similar (*p* > 0.05) in both varieties. As regards glucose 6-P and sucrose, no statistically significant differences were found between control and UV-B plants of both varieties (Figure 5A,B, also confirmed by the ANOVA test). Although the average values fluctuated in the various cases analyzed, they were all part of a physiological fluctuation.

**Figure 6:**
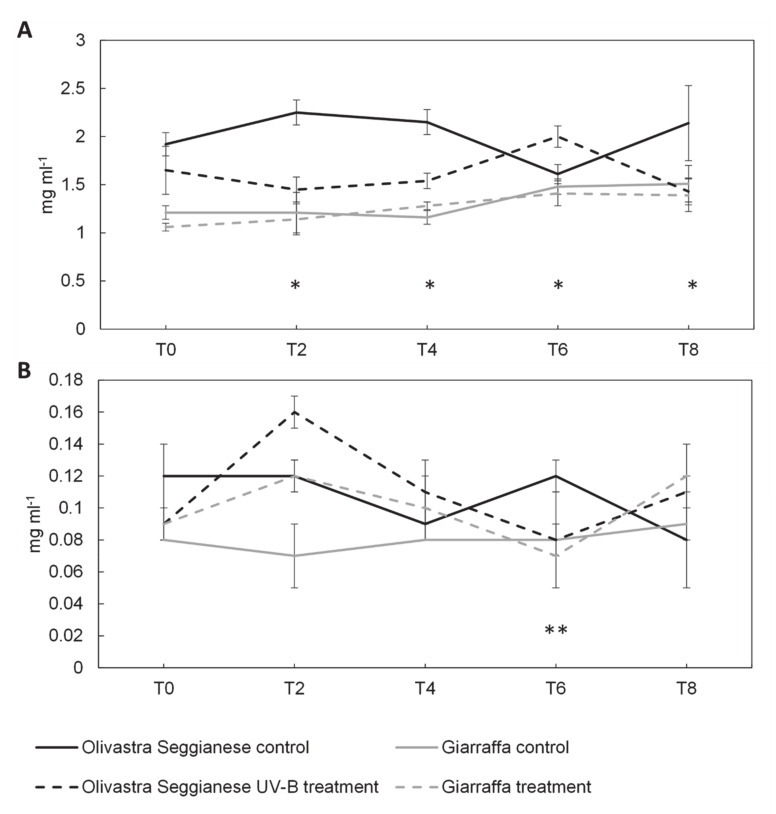
On the x-axes the time points, on the y-axes the concentration of sugars expressed in mg mL^−1^. (**A**) Glucose content in the two olive varieties, Olivastra Seggianese and Giarraffa, under control and UV-B treatment in the different sampling times. For each column, values are given as mean ± standard deviation. Asterisk (*) represent significant differences between control and treated plants of Olivastra Seggianese (*p* ≤ 0.05). (**B**) Fructose content in the two olive varieties, Olivastra Seggianese and Giarraffa, under control and UV-B treatment in the different sampling times. For each column, values are given as mean ± standard deviation. Double asterisk (**) represent significant differences between control and treated plants of Olivastra Seggianese (*p* ≤ 0.01).

The levels of glucose (Figure 6A) in the control and UV-B Giarraffa plants were similar (*p* > 0.05). In fact, the ANOVA test did not give significant results. On the contrary, in the case of Olivastra Seggianese there were significant differences between control and UV-B plants, as confirmed by the ANOVA test. In fact, at the T2, T4 and T8, UV-B plants showed levels of glucose lower than control plants. At time point 6, UV-B plants showed a glucose level higher (*p* ≤ 0.05) than the control.

Concerning the content of fructose, we found that fluctuations for the Olivastra Seggianese variety were statistically significant while those for the Giarraffa variety were not (data confirmed by the ANOVA test); at time point 2 the levels of this sugar in UV-B plants of both varieties were higher than in control plants (Figure 6B). Moreover, at time point 6, the UV-B Olivastra Seggianese plants showed a content of fructose significantly lower than the control ones (*p* ≤ 0.01).

**Figure 7:**
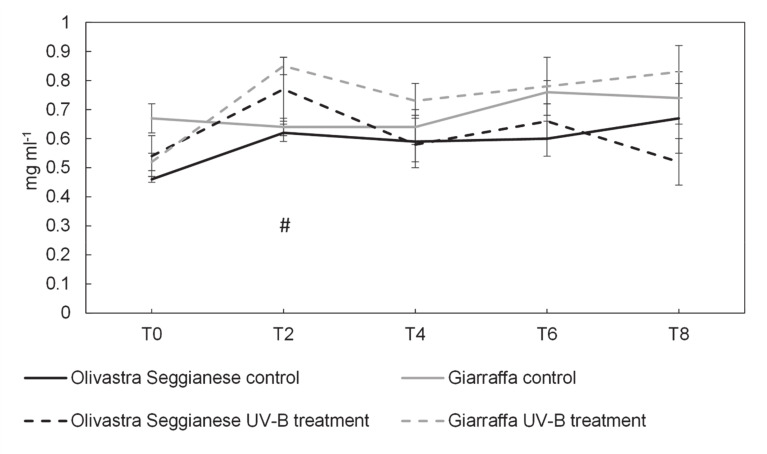
On the x-axes the time points, on the y-axes the concentration of mannitol expressed in mg mL^−1^. Mannitol content in the two olive varieties, Olivastra Seggianese and Giarraffa, under control and UV-B treatment in the different sampling times. For each column, values are given as mean ± standard deviation. Hashtag (#) represent significant differences between control and treated plants of Giarraffa (*p* ≤ 0.05).

Results of mannitol (Figure 7) showed that the concentration of this alcohol-sugar increases in treated plants of both varieties significantly compared to the controls (as confirmed by the ANOVA). The content of mannitol in Giarraffa variety at the T2 point was significantly different between control and treated plants. In fact, mannitol in UV-B plants was 0.85 mg mL^−1^ while in control was 0.64 mg mL^−1^ (*p* ≤ 0.05) (Figure 7). Furthermore, UV-B Giarraffa plants maintained high mannitol concentrations throughout the treatment, unlike UV-B Seggianese plants which resumed the control values after the peak at T2.

### 2.4. Antioxidant Capacity, Polyphenols and Flavonoids

**Figure 8:**
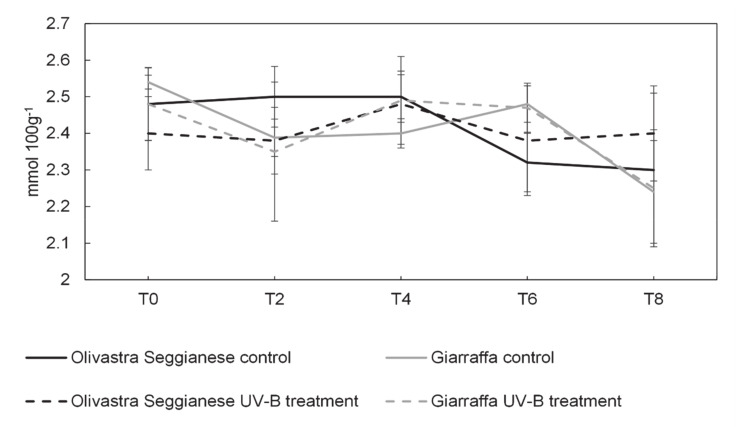
On the x-axes the time points, on the y-axes the concentration of antioxidant expressed in mmol 100 g^−1^. Antioxidant capacity in the two olive varieties, Olivastra Seggianese and Giarraffa, under control and UV-B treatment in the different sampling times. For each line, values are given as mean ± standard deviation.

Figure 8 shows the antioxidant capacity of olive leaves of both varieties (control and UV-B treated plants). No significant differences were found in the content of antioxidants between Olivastra Seggianese and Giarraffa control and UV-B plants throughout the experiment duration.

**Figure 9:**
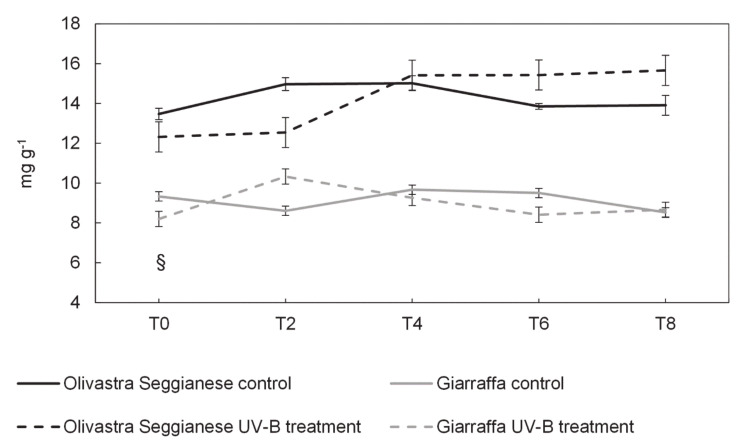
On the x-axes the time points, on the y-axes the concentration of polyphenols expressed in mg g^−1^. Polyphenols content in the two olive varieties, Olivastra Seggianese and Giarraffa, under control and UV-B treatment in the different sampling times. For each line, values are given as mean ± standard deviation. The symbol § represent significant differences between Olivastra Seggianese plants and Giarraffa plants (*p* ≤ 0.05).

Concerning polyphenols, a clear difference (*p* ≤ 0.05) in the total content between the two varieties was observed (Figure 9). In fact, at T0 all plants of Olivastra Seggianese showed an average value of polyphenols of about 13 mg g^−1^ FW, while plants of Giarraffa had a value of about 9 mg g^−1^ FW. Despite this difference, controls of both varieties show a similar trend, particularly after T4. On the contrary, UV-B plants of both varieties show a different trend of response. In fact, UV-B Olivastra Seggianese plants, when compared to control, exhibited a slight increase (*p* > 0.05) in total polyphenols at T4, with an average content of 15.42 mg g^−1^ FW that was followed by a plateau phase until T8. Increase of polyphenols in UV-B Giarraffa plants, as compared to controls, is much more evident from T0 to T2, reaching an average content of 10.33 mg g^−1^ FW, subsequently plants decreased slightly until T8 with polyphenols content values similar (*p* > 0.05) to those of controls.

**Figure 10:**
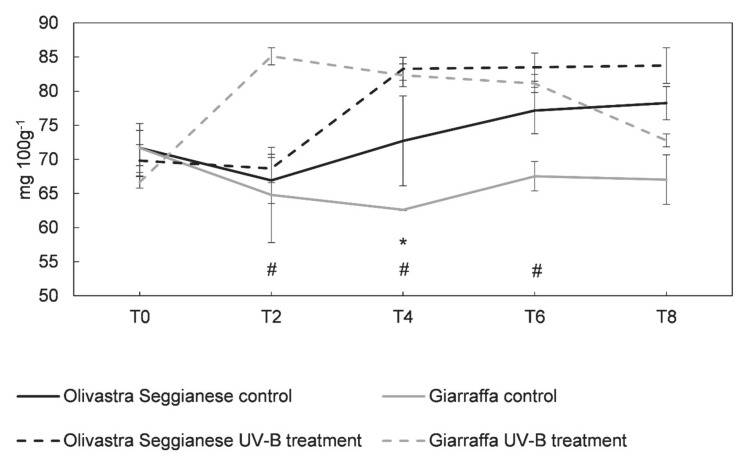
On the x-axes the time points, on the y-axes the concentration of flavonoids expressed in mg 100 g^−1^. Flavonoids content in the two olive varieties, Olivastra Seggianese and Giarraffa, under control and UV-B treatment in the different sampling times. For each line, values are given as mean ± standard deviation. Asterisk (*) represent significant differences between control and treated plants of Olivastra Seggianese (*p* ≤ 0.05). Hashtag (#) represent significant differences between control and treated plants of Giarraffa (*p* ≤ 0.05).

Figure 10 shows the total flavonoids present in the leaves of both varieties (control and UV-B treated plants). The data obtained showed that at T0 the content of flavonoids in plants of the two varieties was very similar (*p* > 0.05) as all plants have an average value of about 70 mg 100 g^−1^ FW. The controls of both varieties showed a similar and linear trend, albeit with some oscillations. On the contrary, UV-B plants of both varieties showed a different trend. In fact, UV-B Olivastra Seggianese plants, compared to the control, showed an increase (*p* ≤ 0.05) in total flavonoids at T4 with an average content of 83.62 mg 100 g^−1^ FW. This increase was followed by a plateau phase lasting until T8, with flavonoid content values similar (*p* > 0.05) to those of controls. Increase of flavonoids in UV-B Giarraffa plants, as compared to controls, is much more evident at T2 (*p* ≤ 0.05), whose average content is 85.11 mg 100 g^−1^ FW. Subsequently Giarraffa plants enter a plateau phase until T6, while maintaining a flavonoid content always higher (*p* ≤ 0.05) than the control. From T6 a decrease in flavonoid content was observed until T8 where the content of flavonoids returned to values similar (*p* > 0.05) to the controls.

## 3. Discussion

Among all physiological processes, photosynthesis is one of the most sensitive to the various stresses that a plant can undergo, especially to the stress induced by high UV-B radiation [31]. In this work, we found that the photosynthetic apparatus of both olive varieties was affected by UV-B. Indeed, photosynthetic efficiency varies over time as stress progresses, comparably with data in the literature [32,33]. More differences are evident when comparing stressed plants with control plants. Before stress (T0), control plants and those to be stressed have *F_v_*/*F_m_* values in the optimal range.

The first symptoms of stress were found already after the second week (T2) of UV-B exposure, particularly in the Giarraffa variety, where the *F_v_*/*F_m_* reach values (<0.75) that are typical of stressed plants [34]. However, after this critical point UV-B Giarraffa plants were able to recover and maintain the levels of photosynthetic efficiency within the optimal range (around 0.8). Contrarily, the Olivastra Seggianese plants were capable to maintain an *F_v_*/*F_m_* value within the optimal range (despite the high variability in the T5), but over the time (T8) showed symptoms of stress. These data suggests that Giarraffa variety are not able to respond immediately in order to preserve the photosynthetic efficiency, but after an adaptive stage triggers a stress protective mechanism allowing the UV-B plants to reestablish the performance and continue to photosynthesize. The Olivastra Seggianese respond earlier but is not able to maintain this capacity over time following the accumulation of the negative effects of UV-B exposure. The pattern of response of the Olivastra Seggianese variety is in line with the one reported by Noguès and Backer [24] in olive plants. These authors reported a decrease of the *F_v_*/*F_m_* to values lower that 0.75 after 14 days of an UV-B BED of 24 KJ m^−2^ d^−1^ (8 h per day). On the contrary, Dias et al. [15] in a Portuguese olive variety exposed to a lower UV-B BED (12.4 KJ m^−2^ d^−1^ for 5 days) found only a small decrease of the *F_v_*/*F_m_* (0.84), unable to compromise the photosynthetic efficiency. In another Mediterranean species, grapevine plants exposed during 60 days to a UV-B BED of 9.6 KJ m^−2^ d^−1^ were able to maintain the *F_v_*/*F_m_* above 0.75 [25]. The degree of damages of UV-B radiation on the PSII functionality seems also to depend on the intensity and duration of exposure as well as the plant species. For instance, according to Albert et al. [35] in some artic plants the UV-B radiation can be considered a source of high stress since it causes a decrease in *F_v_*/*F_m_* and a progressively increasing damage on the photosystem II. 

The pattern of response of Pi suggests that UV-B conditions reduce the absorption, capture and conversion of excitation energy in electron transport [36], being the UV-B Olivastra Seggianese more affected at the end of experiment. As observed for the *F_v_*/*F_m_*, after the first week of UV-B exposure (T2), the Pi was more affected. As also demonstrated in other studies, Pi seems to be more sensitive to environmental stresses than *F_v_*/*F_m_* [37]. The decrease of Pi, as found here for both varieties, is in line with what reported in the literature for other species (maize, sorghum, amaranth and cotton) exposed to high UV-B radiation [9,38].

Taken into account the profiles of *F_v_*/*F_m_* and Pi, we hypothesize that Giarraffa can trigger defense mechanisms suitable for long-lasting UV-B stress unlike Olivastra Seggianese. Comparing the maps of UV index in the various Italian regions [39] and in the area of origin of the two varieties, Giarraffa is widely cultivated throughout southern Italy, where the UV index is higher than in Tuscany, the region of origin of Olivastra Seggianese. Possibly the Sicilian variety would adapt over time to growth and survive in environments with higher UV radiation, so that Giarraffa is better equipped to respond to a prolonged UV-B stress.

There are few studies on the photosynthetic pigments of *O. europaea* and on their change following UV-B stress. UV-B radiation can cause variations in the levels of chlorophyll between 10-70% in plants of agricultural interest [40,41,42,43,44] depending on the species and the intensity of applied stress agent. Chlorophylls are one of most abundant pigments in plant chloroplasts and they are vital to absorb sunlight for photosynthesis. Carotenoids, besides their function as accessory light-harvesting pigments, also act as antioxidants protecting chlorophylls from photooxidation [9]. Within carotenoids, lutein is found mainly in antenna complexes and β-carotene can be found mostly in the reaction centers [45,46]. In the Olivastra Seggianese variety, UV-B treatment seems not to affect the response profile of chlorophyll a, chlorophyll b, β-carotene and lutein for all the various time points analyzed. However, in Giarraffa variety UV-B seems to reduce accumulation of pigments (chlorophylls and β-carotene), particularly after a prolonged period of UV-B exposure (T6). This suggest an adaptation mechanism triggered in Giarraffa that aims to reduce energy absorption and therefore defending against excessive UV-B radiation [47]. Moreover, a reduction of pigments content can also represent a degradation by UV-B radiation as suggested for *Oryza sativa*, *Prunus dulcis* and *Bryum argenteum* [48,49,50]. In *Eucalyptus globulus* [51] and in olive plants [15] exposed to a UV-B BED of around 6 and 12 KJ m^−2^ d^−1^, respectively, the increase of ROS was associated to pigment decrease in UV-B treated plants. 

Abiotic stresses can induce fluctuations in carbohydrates levels due to changes in CO_2_ assimilation, in source-sink carbon partitioning and in the activity of enzymes related to sugars synthesis [52]. In the present work, the soluble sugars glucose and fructose were the most responsive to UV-B treatment. UV-B Olivastra Seggianese plants tend to accumulate less glucose, particularly after the second week, possible due to a reduction of the photosynthetic processes and to a higher use of this sugar to maintain cellular respiration, to counteract the stress conditions or even to restore/increase the levels of other reserve sugars (e.g., starch) or polyols (e.g., mannitol, that tend to increase at T2) [52,53,54]. In turn, UV-B conditions seems, in general, to promote fructose accumulation (except at T6), more markedly in Olivastra Seggianese variety. Fructose increase can result from sucrose degradation as a response to stress or it can provide the substrate to secondary metabolites synthesis (e.g., lignin and phenolic compounds) [52]. Dias et al. [15] reported that olive plants treated with a lower UV-B dose (12 KJ m^−2^ d^−1^) produced less sucrose and starch but maintained glucose and sorbitol contents. Also, in eucalyptus plants, UV-B treatment (BED of 6 KJ m^−2^ d^−1^) decreased the pool of starch and soluble sugars [51]. These authors argued that UV-B can induce starch degradation to provide more soluble sugars necessary to continue plant metabolic activities and to counteract the stress condition. Contrarily, moringa plants treated with a total UV-B dose of 26 KJ m^−2^ showed high functional plasticity increasing soluble sugars, but not changing starch levels [55]. Given the key role of sucrose [56,57,58], we may assume that plants under UV-B stress implement mechanisms to maintain constant sucrose levels and related metabolic processes. However, this is not always the case because sucrose content in leaves of *Eriophorum russeolum* decreased as a result of UV-B stress [59] while fructose and glucose concentration showed no significant decreases.

Mannitol is produced in large quantities and accumulated in the leaves of olive trees [60] as well as in many other plants [61,62]. Like sucrose, it is transported in non-photosynthesizing tissues of plants [63,64,65]; together with glucose, mannitol contributes to the osmotic potential and thus to cell turgor [66] and plays an important role in the response to salt and drought stress [67,68]. In addition to osmotic regulation, mannitol increases scavenging of OH-radicals by stabilizing the structure of macromolecules [69]. The concentration of mannitol increases significantly in both olive varieties analyzed. In Giarraffa at T2 the concentration of mannitol increases in plants subjected to UV-B compared to control and this variety maintains high concentrations of mannitol throughout the treatment compared to Seggianese; the latter does not show critical differences between control and stressed plants. Mannitol concentration may increase in response to UV-B stress for an osmoprotective and antagonistic function against free radicals [70]. Since Giarraffa responds better than Seggianese to UV-B stress and has a higher concentration of mannitol, it probably developed this response mechanism to adapt to the more intense radiation in its area of origin.

High UV-B radiation can trigger an increase of reactive oxygen species (ROS) at cell level, which cause oxidation of proteins, lipids and other biomolecules, thus compromising the entire cellular functioning [71]. To deal with the damage caused by ROS, living organisms have developed a complex defense system consisting of enzymatic and non-enzymatic antioxidants [72]. The antioxidant capacity gives a general information about the antioxidant levels [73] and in the Olivastra Seggianese and Giarraffa both control and UV-B plants respond very similar. Polyphenols play an important role in *O. europaea* oxidative stress control and antioxidant responses against abiotic stress, such as UV-B radiation, drought and heat [23,29]. Olive leaves contain a large variety of phenolic compounds, such as flavonoids (e.g., luteolin-7-*O*-glucoside, luteolin-5-*O*-glucoside, luteolin-4-*O*-glucoside, quercetin-7-*O*-rutinoside, quercetin-3-*O*-glucoside, apigenin-7-*O*-glucoside and chrysoeriol-7-*O*-glucoside), secoiridoids (e.g., oleuropein), hydroxycinnamic acid derivatives (e.g., verbascoside), phenolic alcohols (e.g., hydroxytyrosol and tyrosol) and phenolic acids (e.g., chlorogenic and caffeic acids) [23,74,75]. The profile of response of total polyphenols showed considerable difference already at T0, which can be attributed to varietal differences. Giarraffa respond first (after the first week) to UV-B radiation increasing polyphenols pools. On the other hand, Olivastra Seggianese plants respond latter to UV-B triggering only an increase of polyphenols up to T2. This response can generally be assumed as an augment of the availability of antioxidant defense compounds [76]. Within the class of polyphenols, the flavonoids are one of the most abundant compounds with antioxidant properties [77]. They are produced in the epidermal layers of leaves and they likely absorb a large portion of incident UV-B radiation reducing the penetration of UV in the lower tissues of leaves [22,78]. Moreover, these secondary metabolites also play an important role as ROS scavengers [79]. Flavonoids, especially the ortho-dihydroxy B-ring substituted flavonoids (e.g., quercetin 3-*O*-glucoside and luteolin 7-*O*-glucosides, commonly found in olive leaves), have an important role in ROS-scavenging. Flavonoids quench the ROS by reducing the singlet oxygen’s, hindering of enzymes involved in ROS generation (lipoxygenase, cyclooxygenase xanthine oxidase, monooxygenase), by chelating transition metal ions which trigger the ROS production, and quenching lipid peroxidation by number of free radical reactions, and help in the recycling of other antioxidants [22,23,77]. Pearson’s [80] correlation coefficient analysis between flavonoid content and Pi and between flavonoid content and *F_v_*/*F_m_*, shows us how in both cases there is a correlation between these two variables. These correlations are negative with r values of −0.712 and −0.749 respectively. A negative relationship indicates that low scores on one variable correspond to high scores on the other variable [80]. That is, in the specific case of this study, the worsening of the health of the plants which results in a decrease in the photosynthetic efficiency values (*F_v_*/*F_m_* and Pi) results in an increase in the flavonoid content. This increase, therefore, could be interpreted as a defense mechanism that plants put in place in order to cope with stress from UV-B radiation. As observed for the total polyphenols, Giarraffa respond first to UV-B stress (during the first weeks), and over time total flavonoids levels tend to decrease. In turn, Olivastra Seggianese respond latter (after the second week) and maintain high levels of these compounds until the end of experiment. These distinct profiles of antioxidant response and also photosynthetic efficiencies to UV-B treatment triggered in the two varieties may be related and may support the hypothesis that Giarraffa is able to activate defense mechanisms already after the first weeks of UV-B stress thereby performing, in a long term, better than Olivastra Seggianese. This higher defense capacity of Giarraffa is also supported by the slight decrease of antioxidants over the second week, which may result from its efficient use to neutralize ROS and therefore protect olive plants from oxidative damage, as already reported in olive trees under UV-B conditions [23]. The importance of polyphenols, particularly the flavonoids, in olive protection against UV-B stress was also highlighted by Noguès and Backer [24].

UV stress as other environmental factors including oxygen shortage or pathogen invasion induces oxidative stress by generation of ROS and the plants defend themselves by the activation of an antioxidants system. Flavonoids may work as ROS scavenging compounds in a cooperative or compensative activity within this complex antioxidant system. All this considered, a trait as a higher production of flavonoids, which this study demonstrated to vary within the cultivars, could be helpful in explaining olive fitness in hostile environment. Breeding of this species will take advantage of any information relative to parental lines to be used for crossing with superior metabolic performances. In our opinion, after our study, Giarraffa could be one of the cultivars to be further analyzed and used to obtain new plants with metabolic features fit to challenge the environmental stresses caused by climate changes.

## 4. Materials and Methods

### 4.1. Plant Growth Conditions

Olive trees (*Olea europaea* L.) of 18 months of two varieties (Olivastra Seggianese and Giarraffa) were taken from the nursery of the “Società Pesciatina di Orticultura” (Pescia, PT, Italy) where the plants were grown in a greenhouse. Subsequently, plants were transferred to climatic cells with the following environmental conditions: temperature: 21 °C; relative humidity (RH): 60%; photoperiod: 14 light h, 10 dark h [81]; light intensity: 500 µmol m^−2^ s^−1^; watering: 400 mL water for each plant once a week; commercial substrate type: “Vigor Plant soil” (Vigor plant Italia srl Fombio, Fombio, Italy).

### 4.2. Application of UV-B Treatment

Ultraviolet radiation was provided by two TL20W/12 lamps (Philips, Milano, Italy) that emit in the wavelength of UV-B rays and that have already been widely used and described in the literature; lamps were prepared and used exactly according to the protocol of Allen et al. [81]. Plants (*n* = 16 for each variety) were positioned under UV-B lamps in the climatic cell. Every day, the homogeneity of the UV-B radiation emitted by the lamps was verified using a Power Meter 840 with Sensor 818-UV (Newport Optical, Irvine, CA, USA). The UV-B biologically effective dose (BED), 25 KJ m^−2^ d^−1^, was calculated according to Correia et al. [82]. Control plants (*n* = 16 for each variety), present in the same climatic cell, have been carefully separated from those treated by means of a plasterboard panel that shielded most of the UV radiation (BED of 1 KJ m^−2^ d^−1^). The UV-B treatment corresponds to a high UV-B dose, but within the natural values already reported in some parts of the earth surface [5].

### 4.3. Determination of Photosynthetic Defficiency

Photosynthetic efficiency has been estimated by induction of chlorophyll fluorescence using a Handy PEA 2000 fluorimeter (Hansatech Instruments, King’s Lynn, Norfolk, UK). Fluorometric analysis of leaf chlorophyll were performed in vivo at ambient temperature, and the changes of the level of fluorescence emission were measured in order to obtain the effectiveness of light use in the photosynthetic process. After 30 min of dark adaptation, the leaf was illuminated for about one second (peak at 650 nm, 3000 µmol m^−2^ s^−1^, an intensity of excitation sufficient to ensure the closure of all PSII reaction centers) and the fluorescence signal was recorded. For each plant (control and stressed), the values of *F_v_*/*F_m_* and PI were collected weekly for 8 weeks in order to identify the time when plants begin to perceive UV-B stress. The following equations were used to calculate *F_v_*/*F_m_* and PI parameters [83] (Equations (1) and (2)):

(1) $$F_{v}/F_{m}=\;\left(F_{m}-F_{0}\right)/F_{m}$$

(2) $$PI_{ABS}=\frac{1-(F_{0}/F_{m})}{M_{0}/V_{j}}\;\times \;\frac{F_{m}-F_{0}}{F_{0}}\;\times \;\frac{1-V_{j}}{V_{j}}$$

where *F_m_* is the maximum fluorescence value, *F*_0_ is fluorescence value at zero instant, *F_v_* is a difference between *F_m_* and *F*_0_, *V_j_* is relative *F_v_*, and *M*_0_ is the initial slope of fluorescence kinetics. *F_v_*/*F_m_*, therefore, represents an index from the maximum value of 1.00, equivalent to 100% of the maximum photochemical efficiency of photosystem II. The performance Index (Pi), a more sensitive parameter indicating the possible variations of the entire photosynthetic apparatus, including photosystems I (PSI) and II (PSII). Pi is a multiparametric expression that considers all the main photochemical processes, such as absorption and capture of excitation energy, transport of electrons over the primary plastoquinone (QA) and dissipation of excess excitation energy.

### 4.4. Analysis of Photosynthetic Pigments

Analysis of the photosynthetic pigments was carried out on frozen leaf samples using high performance liquid chromatography technique (HPLC—Waters LC Module One, Waters S.p.A., Milano, Italy) following the method of Suzuki et al., [84]. Olive leaves were powdered with liquid nitrogen, approximately 20 mg of each leaf sample was mixed in Eppendorf tubes with 1 mL of ethanol. Subsequently, samples were homogenized by Ultra-turrax (IKA^®^-Werke GmbH & Co. KG, Staufen im Breisgau, Germany) for about 2 min until complete rupture of cells. The homogenate was centrifuged at 13,000× *g* for 5 min at 4 °C. After that, supernatants containing pigments were transferred to a glass test tube. Then 20 μL aliquots of sample were injected into the HPLC column. The column used was a C18 (25 cm × 4.6 mm, grain size 5 μm). The mobile phase is a ternary mobile phase with the following gradient conditions (Table 1):

The chromatographic run was carried out at a flow of 1 mL min^−1^, room temperature; the eluate was monitored at the wavelength of 440 nm and the separation time was 30 min. The following reference pigments have been used: xanthophyll (lutein) 10 μg mL^−1^ (elution time 17.59 min); trans β-carotene 50 μg mL^−1^ (elution time 37.49 min); chlorophyll a 10 μg mL^−1^ (elution time 25.23 min); chlorophyll b 10 μg mL^−1^ (elution time 21.7 min). Identification of the various components was obtained by programming the integrated UV detector with specific excitation wavelengths (440 nm) by comparing the retention times with those of reference standards and by comparing the characteristics of the absorption spectra of individual chromatographic fractions with those found in the literature. Subsequently, the concentrations of the 4 pigments were determined through the CSW-32 analysis software (Clarity—Data APEX, Prague, The Czech Republic) calculating each peak area. The protocol was repeated three times for each sample.

### 4.5. Analysis of Sugars

Analysis of sugars (sucrose, fructose, glucose, glucose 6-phosphate and mannitol) was conducted by HPLC. Approximately 100 mg of leaf samples were first powdered with liquid nitrogen and then supplemented with 1 mL of water in 2 mL Eppendorf tubes. Subsequently, samples were homogenized using the Ultra-turrax homogenizer for about 2 min until complete rupture of cells. The homogenate was subjected to centrifugation at 3000× *g* for 5 min, the supernatants transferred to 2 mL Eppendorf tubes and then centrifuged again at 12,000× *g* for 5 min. Samples were filtered (0.45 µm) and about 20 µL of each extract was injected and examined using a Waters Sugar-Pak I ion exchange column (6.5 × 300 mm) at a temperature of 90 °C. The mobile phase consists of MilliQ water (pH 7) with a flow of 0.3 mL min^−1^. The overall duration of the separation was 30 min. The elution times of sugars are as follows: glucose 6-P—about 5 min; sucrose—about 8 min; glucose—about 10 min; fructose—about 11 min; and mannitol—about 13 min. Identification of the components was obtained using a Waters 2410 refractive index detector, by comparing the retention times with those of reference standards. For each peak, the retention factor allows to identify the type of eluted molecule, while the curve area is proportional to the quantity. The protocol was repeated three times for each sample.

### 4.6. Determination of the Antioxidant Capacity, Polyphenols and Flavonoids

Frozen leaves (1 g) were macerated with 3 mL of 70% acetone. Subsequently, samples were homogenized with a Miccra rt homogenizer (IKA^®^-Werke GmbH & Co. KG, Staufen im Breisgau, Germany) for about 2 min, and then inserted in a sonicator for 20 min for the complete breakage of cellular components. The homogenate was centrifuged at 4000× *g* for 5 min at 4 °C. Then, the supernatants were taken and used for analysis.

#### 4.6.1. Ferric Ion Reducing Antioxidant Power—FRAP

For determination of total antioxidants, each reaction tube contained 2040 μL of acetate buffer, 200 μL of 2,4,6-Tri(2-pyridyl)-s-triazine (TPTZ), 200 μL of ferric chloride and 20 μL of leaf extract. Subsequently, samples were placed at 37 °C for 60 min. After incubation, samples were read at a wavelength of 593 nm. The antioxidant content was calculated based on a calibration curve of standard solutions of ferrous sulphate. The experiment was conducted in triplicate for each sample.

#### 4.6.2. Folin-Ciocalteu Method for the Determination of Total Polyphenols

Each reaction tube contained 500 μL of leaf extract, 3000 μL of distilled water, 250 μL of FC reagent, 750 μL of sodium carbonate (Na_2_CO_3_) and 950 μL of distilled water. Subsequently, samples were placed at 37 °C for 30 min. After the incubation, samples were read at 765 nm. Polyphenols content was calculated based on a calibration curve of standard solutions of gallic acid. The experiment was conducted in triplicate for each sample.

#### 4.6.3. Aluminum Chloride Method for the Determination of Total Flavonoids

Each reaction tube contained 500 μL of leaf extract, 1.5 mL of 95% ethanol, 100 μL of aluminum chloride, 100 μL of potassium acetate and 2.8 mL of distilled water. Samples were maintained at room temperature for 30 min, and then were read at 415 nm. Total flavonoids were determined based on a calibration curve of standard solutions of quercetin. The experiment was conducted in triplicate for each sample.

### 4.7. Statistical Analysis

In order to verify the significance of the data obtained, the ANOVA test of the two-factor variance with replication and the *t*-test (* *p* < 0.05, ** *p* < 0.01) were carried out. To verify the correlation between the performance index and the flavonoid content and between *F_v_*/*F_m_* and the flavonoid content, the Pearson correlation coefficient was carried out. ANOVA and the Pearson correlation coefficient were performed by Systat 11 statistical package (Systat Software Inc., Richmond, CA, USA).

## 5. Conclusions

Given the high and multiple importance of the olive tree, it is essential to study its responses to stressful agents, such as excessive UV-B radiation, in order to understand the defense mechanisms and identify the most resistant varieties. This study confirms that UV-B radiation is a dangerous source of stress for olive tree, especially in today’s increasingly changing environmental conditions. Although the two varieties showed symptoms of UV-B stress and activate antioxidant defence mechanisms, they exhibited evident different response patterns and timescales. The T2 could be the critical stage, since around this time point started to be more notorious the stress symptoms (e.g., reduction of *F_v_*/*F_m_*) and antioxidant defenses are activated. Giarraffa variety seems better suited to prolonged UV-B stress, possible due to a more efficient and quick activation of the antioxidant response (e.g., flavonoids use to counteract ROS) and due to its capacity to maintain the photosynthetic efficiency as well as a relatively higher content of mannitol. Moreover, pigments reduction after a long period of UV-B exposure can also be an adaptation mechanism triggered by Giarraffa to reduce energy absorption under UV-B stress. Olivastra Seggianese seems less suited to overcome UV-B stress for a long period (e.g., higher reduction of *F_v_*/*F_m_*) and has a higher necessity to use sugars (e.g., glucose) possible to counteract stress and to restore energy.

## Figures and Tables

**Table 1:** Gradient values used in HPLC analyses for pigment separation.

Time (min.)	% A	% B	% C
Initial	75	25	0
4	75	25	0
5	100	0	0
11	80	0	20
20	65	0	35
30	75	25	0

Solvent A: methanol; Solvent B: water; Solvent C: acetone.
